# Ascorbic Acid Protects Bone Marrow from Oxidative Stress and Transient Elevation of Corticosterone Caused by X-ray Exposure in *Akr1a*-Knockout Mice

**DOI:** 10.3390/antiox13020152

**Published:** 2024-01-25

**Authors:** Tomoki Bo, Hidekazu Nohara, Ken-ichi Yamada, Satoshi Miyata, Junichi Fujii

**Affiliations:** 1 Laboratory Animal Center, Institute for Promotion of Medical Science Research, Yamagata University Faculty of Medicine, 2-2-2 Iidanishi, Yamagata 990-9585, Japan; 2 Physical Chemistry for Life Science Laboratory, Faculty of Pharmaceutical Sciences, Kyushu University, 3-1-1 Maidashi Higashi-ku, Fukuoka 812-8582, Japan; kenyamada@phar.kyushu-u.ac.jp; 3 Miyata Diabetes and Metabolism Clinic, 5-17-21 Fukushima, Fukushima-ku, Osaka 553-0003, Japan; 4 Department of Biochemistry and Molecular Biology, Graduate School of Medical Science, Yamagata University, 2-2-2 Iidanishi, Yamagata 990-9585, Japan

**Keywords:** reactive oxygen species, adrenal, adrenocorticotropic hormone

## Abstract

Bone marrow cells are the most sensitive to exposure to X-rays in the body and are selectively damaged even by doses that are generally considered permissive in other organs. Ascorbic acid (Asc) is a potent antioxidant that is reported to alleviate damages caused by X-ray exposure. However, rodents can synthesize Asc, which creates difficulties in rigorously assessing its effects in such laboratory animals. To address this issue, we employed mice with defects in their ability to synthesize Asc due to a genetic ablation of aldehyde reductase (*Akr1a*-KO). In this study, concentrations of white blood cells (WBCs) were decreased 3 days after exposure to X-rays at 2 Gy and then gradually recovered. At approximately one month, the recovery rate of WBCs was delayed in the *Akr1a*-KO mouse group, which was reversed via supplementation with Asc. Following exposure to X-rays, Asc levels decreased in plasma, bone marrow cells, and the liver during an early period, and then started to increase. X-ray exposure stimulated the pituitary gland to release adrenocorticotropic hormone (ACTH), which stimulated corticosterone secretion. Asc released from the liver, which was also stimulated by ACTH, appeared to be recruited to the bone marrow. Since corticosterone in high doses is injurious, these collective results imply that Asc protects bone marrow via its antioxidant capacity against ROS produced via exposure to X-rays and the cytotoxic action of transiently elevated corticosterone.

## 1. Introduction

Individuals exposed to radiation from either a nuclear accident or during a medical treatment are at risk of developing acute radiation syndrome, which characteristically occurs in hematopoietic, gastrointestinal, neurovascular, and cutaneous systems [1,2]. Reactive oxygen species (ROS) produced upon exposure to X-rays are major components of tumoricidal action during radiation therapy and are known to oxidatively damage normal tissues [3]. Because bone marrow cells (BMCs) and white blood cells (WBCs), notably lymphocytes and granulocytes, are extremely sensitive to X-ray exposure [4,5], even otherwise permissive doses of X-rays may disrupt such immune systems, thereby creating a disproportionate susceptibility to infection. While oxygen concentrations largely affect the susceptibility of cells to radiation therapy [6], other humoral factors are also known to be involved in the sensitivity to and/or the protection against exposure to X-rays. Compounds that exhibit antioxidant activity are promising agents that tend to reduce oxidative damage to BMCs. Ascorbic acid (Asc) is condensed in some cellular organelles and is considered to be one of the strongest forms of antioxidants among nutritional compounds [7,8]. In fact, many studies have established the beneficial action of Asc in protecting cells from unfavorable reactions caused by exposure to X-rays [9,10,11,12]. The protective effects of Asc appear to be evident following low doses of irradiation, which is not the case following high doses [13].

Most animals are able to synthesize Asc, mainly in the liver, and this includes rodents [14,15]. The gene encoding L-gulono-γ-lactone oxidase (*Gulo*) that catalyzes the last step in the synthesis of Asc was, however, likely mutated approximately 63,000,000 years ago, rendering primates unable to synthesize it [16]. Two members of the aldo-keto reductase (AKR) superfamily, aldehyde reductase (AKR1A) and aldose reductase (AKR1B), catalyze the reductive conversion of D-glucuronate into L-gulonate in the Asc synthesis pathway and contribute 85–90% and 10–15% to total Asc synthesis, respectively [17,18]. *Akr1a*-knockout (*Akr1a*-KO) mice die within one year when growing while being fed an Asc-deficient diet, but supplementation with Asc (1.5 mg/mL) in drinking water extends their lifespan to an extent similar to that of wild-type (WT) mice [19]. In the meantime, *Akr1a*-KO mice show an increase in plasma levels of corticosterone, aggressive behavior and developmental retardation [20,21]. Asc is abundantly present in the adrenal glands [22] and is thought to stimulate steroidogenesis in the adrenal glomerular zone [23,24]. Nevertheless, Asc reportedly suppresses corticosterone secretion [25]. Cells take up Asc via sodium ascorbate co-transporters, SVCT1 and SVCT2, from nutrients in the intestine and in blood [26]. The ablation of *SVCT2* decreases catecholamine storage in the adrenal medulla and plasma levels of corticosterone [27]. Although developmental retardation is associated with high levels of corticosterone and low Asc concentrations [21], supplementation with Asc affects neither aggressive behavior nor high corticosterone levels in *Akr1a*-KO mice [20].

Exposure to X-rays stimulates pituitary glands to release adrenocorticotropic hormone (ACTH), which then stimulates adrenal glands to release corticosterone and also Asc [28,29,30]. In the meantime, X-ray exposure decreases Asc in the BMCs of rats [31]. For the clinical application of Asc, it is essential to understand the regulatory mechanism of Asc concentrations in BMCs in order to maintain the physiological relevance between Asc and the endocrine system during radiation therapy. To gain insights into these issues, we employed *Akr1a*-KO mice and WT mice that were exposed to non-lethal, permissive doses (2 Gy) of X-rays in this study.

## 2. Materials and Methods

### 2.1. Animals

*Akr1a*-KO mice with a C57BL/6 background were obtained using a gene-targeting technique [18], bred in our institution, and used throughout this study. The female WT and *Akr1a*-KO mice were weaned at 30 days of age and fed a standard diet without Asc (Picolab 5053, LabDiet, St. Louis, MO, USA) ad libitum with free access to either water or water containing 1.5 mg/mL of Asc (FUJIFILM Wako Pure Chemical, Osaka, Japan) until they were used. The animals used were at least 10 weeks old. Animal experiments were performed in accordance with the Declaration of Helsinki under protocols approved by the Animal Research Committee at Yamagata University.

### 2.2. X-ray Irradiation

During exposure to X-rays, the mice were anesthetized and immobilized. X-ray irradiation at 2 Gy was performed using a Shimadzu X-TITAN 225S X-ray generator (Shimazu, Kyoto, Japan) at 200 kV and 14 mA with a 1.0 mm aluminum filter.

### 2.3. Blood Cell Counts in Mice

Following exposure to X-rays, 100 µL of mouse blood was collected in EDTA tubes after 3 days and again every 7 days from the submandibular vein using 5 mm Goldenrod Animal Lancet (MEDIpoint, New York, NY, USA). Blood samples were analyzed with a hematology analyzer, VETSCAN HM5 (ABAXIS, Union City, CA, USA), to acquire lymphocytes and WBCs.

### 2.4. BMC Isolation

In order to isolate the BMCs, WT and *Akr1a*-KO mice were euthanized by cervical dislocation at indicated times following exposure to X-rays, and their femurs were removed. All bone marrow was flushed out with PBS and filtered using a 70 µm Cell Strainer (Corning, NY, USA). Following centrifugation, erythrocytes were lysed with 1 mL of RBC lysis buffer (155 mmol/L NH_4_Cl, 10 mmol/L KHCO_3_ and 0.1 mmol/L EDTA). Following centrifugation, the supernatant was aspirated. The BMCs were frozen with liquid nitrogen until use. Data using these cells were expressed per protein amount.

### 2.5. Measurement of the Reduced Form of Asc

To measure Asc levels, plasma, liver, adrenal glands and BMCs were collected and used for the experiments following exposure to X-rays. The tissue samples (10 mg) excluding plasma were homogenized with a glass–Teflon homogenizer in 100 µL of lysis buffer (50 mmol/L Tris-HCl, pH 7.5, 1% (*v*/*v*) Triton X-100, 5% (*v*/*v*) glycerol, 5 mmol/L EDTA, and 150 mmol/L NaCl). Samples were incubated with 50 µmol/L of Naph-DiPy provided by Dr. Ken-ichi Yamada [32] for 30 min at room temperature, and the fluorescence intensity was measured using a microplate reader, Varioskan Flash (Ex: 310 nm, Em: 410 nm; Thermo Fisher Scientific, Waltham, MA, USA). The protein concentration of the samples was measured using Bio-Rad Protein Assay Dye Reagent (Bio-Rad, Hercules, CA, USA). The results were corrected per unit of protein.

### 2.6. Measurement of Blood Biochemical Parameters

Blood (100 µL) was collected from the submandibular vein using 5 mm Goldenrod Animal Lancet on the first day following exposure to X-rays and again 3 days later. Following centrifugation at 800× *g* for 5 min at 4 °C, plasma was collected. Blood biochemical parameters were measured using DRI-CHEM NX500V (FUJIFILM Wako Pure Chemical) in accordance with the manufacturer’s protocol. The following parameters were measured: blood urea nitrogen (BUN), creatinine (CRE), aspartate aminotransferase (AST), and alanine aminotransferase (ALT).

### 2.7. Measurements of ACTH and Corticosterone

Blood was collected from mice at the indicated time points following exposure to X-rays. Following centrifugation at 800× *g* for 5 min at 4 °C, plasma was collected; then, ACTH and corticosterone levels were measured using an ACTH ELISA kit (M046006; MDB, Zürich, Switzerland) and Corticosterone EIA Kit (YK240; Yanaihara Institute Inc., Shizuoka, Japan), respectively, in accordance with the manufacturer’s protocol. To measure corticosterone levels in the adrenal grands, the glands were homogenized in lysis buffer. Following centrifugation at 18,000× *g* for 15 min at 4 °C, supernatants were collected and used for corticosterone measurement. The protein concentration of the samples was measured using Bio-Rad Protein Assay Dye Reagent. The results were corrected per unit of protein.

### 2.8. Western Blotting

Tissue samples were homogenized with a glass–Teflon homogenizer in lysis buffer. After centrifugation at 18,000× *g* for 15 min at 4 °C, supernatants were collected. A 3-fold-concentration of Laemmli’s sample buffer (0.1875 mol/L Tris-HCl, pH 6.8, 15% (*v*/*v*) β-mercaptoethanol, 6% (*w*/*v*) SDS, 30% (*v*/*v*) glycerol, and 0.006% (*w*/*v*) bromophenol blue) was added to the supernatants, and the samples were boiled for 3 min. Proteins were separated via SDS–PAGE and transferred onto a nitrocellulose membrane (Advantec TOYO, Tokyo, Japan). The membrane was blocked with TBST (10 mmol/L Tris-HCl, pH 7.4, 0.1 mol/L NaCl, and 0.1% Tween-20) containing 5% (*w*/*v*) nonfat skim milk and probed with specific antibodies diluted with TBST containing 5% (*w*/*v*) nonfat skim milk overnight at 4 °C. After probing with HRP-conjugated secondary antibodies, the bound antibodies were detected using a Western Lightning Plus-ECL substrate (Merck Millipore, Burlington, MA, USA). Image acquisition was performed using an image analyzer (ImageQuant LAS500, GE Healthcare, Chicago, IL, USA), and image analysis was performed using ImageJ software version 1.54f. The following antibodies were used in this study: StAR (Santa Cruz Biotechnology, Santa Cruz, CA, USA), Actin (Santa Cruz Biotechnology), P450_scc_ (Proteintech, Rosemont, IL, USA), AKR1A [33], mouse anti-rabbit IgG-HRP (Santa Cruz Biotechnology), and m-IgGκ BP-HRP (Santa Cruz Biotechnology). The dilution ratios for the primary and secondary antibodies are 1:1000 and 1:2000, respectively.

### 2.9. Quantitative Reverse-Transcription Polymerase Chain Reaction (RT-PCR)

RNA from mouse livers (20 mg) and BMCs (10 mg) were purified using ISOGEN II (Nippongene, Tokyo, Japan). Total RNA concentration was measured using BMe-spect2 (Malcom, Tokyo, Japan), and cDNA was prepared from 500 ng of RNA using a primescript cDNA synthesis kit (TaKaRa, Shiga, Japan). Amplification of the cDNAs using the corresponding primers (Supplementary Table S1) followed by separation on agarose gels was performed. Quantitative RT-PCR analyses were performed using the CFX96 real-time PCR system (Bio-Rad) and a Thunderbird SYBR qPCR mix (TOYOBO, Osaka, Japan) in accordance with the manufacturer’s recommendations. The relative mRNA levels of *SVCT1* and *SVCT2* were calculated using the ΔΔCt method with normalization to the level of HPRT1, which was used as the internal control.

### 2.10. Adrenalectomy (ADX)

Mice were anesthetized using a mixture of 0.3 mg/kg of medetomidine, 4 mg/kg of midazolam, and 5 mg/kg of butorphanol. The left sides of the dorsal skin and peritoneum were sequentially incised. Then, the adrenal gland was gripped with forceps and cut off using scissors. The peritoneum and skin were sequentially stitched. The right-side operation followed the same procedure as that on the left side. The animals were used in the experiment at least 2 weeks following surgery.

### 2.11. Cultivation of BMCs and Lactate Dehydrogenase (LDH) Release Assay

BMCs were isolated from WT mice and were incubated in RPMI1640 culture media (Thermo Fisher Scientific) containing 10% FBS (Sigma Aldrich, St. Louis, MO, USA) and 50 U/mL of penicillin and streptomycin (FUJIFILM Wako Pure Chemical) overnight. When necessary, the BMCs were treated with 50 µmol/L Asc or 200 nmol/L dexamethasone (FUJIFILM Wako Pure Chemical) 2 h before X-irradiation. One day, following the exposure to X-rays, the medium containing BMCs was collected and centrifuged at 15,000× *g* for 5 min at 4 °C. The supernatant was subjected to Cytotoxicity LDH Assay Kit-WST (Dojindo, Kumamoto, Japan) in accordance with the manufacturer’s instructions.

### 2.12. Statistical Analysis

All results are expressed as the mean ± the standard error (SE) of experiments performed in triplicate at least. Comparisons between the two groups were performed using Student’s *t*-test. For multiple comparisons, the Tukey–Kramer test was used. The minimum level of significance was set at *p* < 0.05.

## 3. Results

### 3.1. Akr1a-KO Delayed the Recovery of WBC Counts following Exposure to X-rays

**Figure 1:**
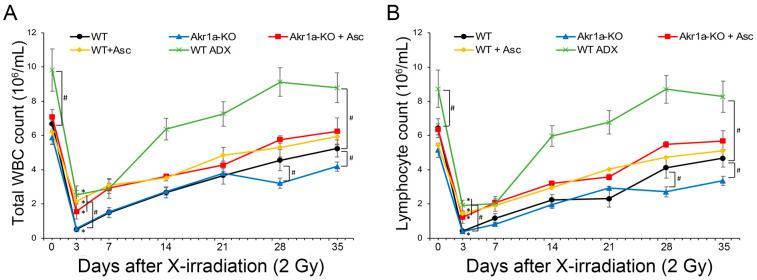
*Akr1a*-KO delayed the recovery of WBCs following exposure to X-rays. Blood samples were analyzed using a hematology analyzer following exposure to X-rays at 2 Gy in WT and *Akr1a*-KO mice with or without Asc. In WT ADX mice, the measurement was begun 2 weeks after adrenalectomy. (**A**) Total WBC counts were measured following exposure. (**B**) Lymphocyte counts were measured after exposure. Data are expressed as the mean ± SE for 5–7 animals per group. * *p* < 0.05 vs. 0 Gy and # *p* < 0.05 vs. WT mice. WBC, white blood cells; WT ADX, mouse with adrenalectomy.

To examine the effect of Asc on the WBC population following exposure to X-rays, we initially measured the blood cells of WT and *Akr1a*-KO mice post-exposure. Following exposure at 2 Gy, WBC counts immediately decreased and then gradually recovered (Figure 1A). Since the majority of WBCs comprise lymphocytes, the radiation-induced decrease in WBCs was attributed to changes in lymphocytes (Figure 1B, Supplementary Figure S1). While there was no difference between WT and *Akr1a*-KO mice 21 days following the initial exposure to X-rays, delayed recovery was observed in the *Akr1a*-KO mice at 28 and 35 days post-exposure. Supplementing Asc significantly alleviated the radiation-induced decrease in WBC counts, and the improving effect of Asc was comparable between both WT and *Akr1a*-KO mice.

Previous studies have reported that corticosterone impairs WBC recovery [34], and we assumed that corticosterone might affect the results of the current study. To investigate the effect of corticosterone on WBC recovery following exposure to X-rays, WT ADX mice were used. The recovery rate of the WBC count post-exposure in WT ADX mice was significantly increased in ADX mice compared with that measured in WT mice. We also measured the blood biochemical parameters associated with liver and kidney function, but exposure to X-rays at 2 Gy induced neither liver nor kidney damage in either WT or *Akr1a*-KO mice (Supplementary Figure S2). These results suggest that the recovery of WBCs following exposure to a permissive dose of X-rays was delayed in *Akr1a*-KO mice, and that the secretion of corticosterone following exposure could have adversely affected the recovery of WBC counts.

### 3.2. X-ray Irradiation Decreased Asc Levels in the Plasma, Liver and BMCs 

**Figure 2:**
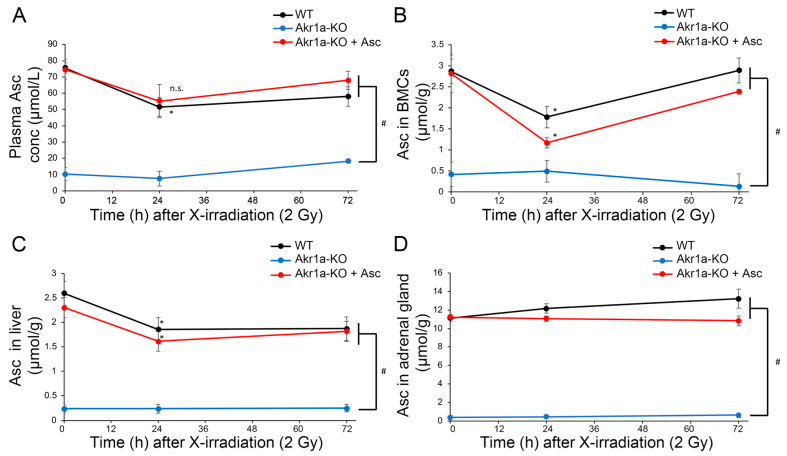
Exposure to X-rays decreasing the Asc levels in plasma, BMCs and liver. Asc levels in plasma (**A**), BMCs (**B**), liver (**C**), and adrenal glands (**D**) were measured using Naph-DiPy in WT and *Akr1a*-KO mice with or without Asc following exposure to X-rays at 2 Gy. The fluorescence intensity was measured using a microplate reader (Ex: 310 nm, Em: 410 nm). Data are expressed as the mean ± SE for 3–5 animals per group. * *p* < 0.05 vs. 0 Gy and # *p* < 0.05 vs. WT mice and *Akr1a*-KO mice supplemented with Asc. n.s., not significant.

**Figure 3:**
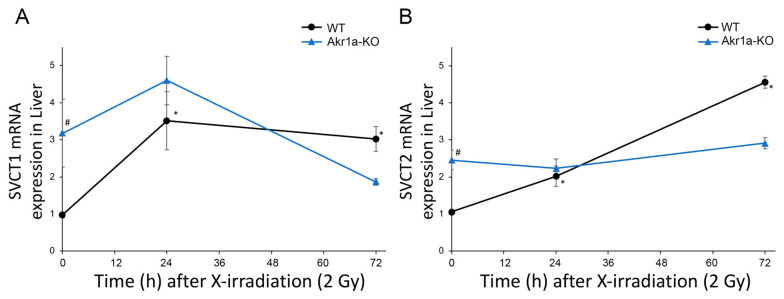
Increased *SVCT1* and *SVCT2* mRNA levels in the liver following exposure to X-rays. The expression of *SVCT1* (**A**) and *SVCT2* (**B**) mRNA in the liver was measured via quantitative RT-PCR following exposure to X-rays in WT and *Akr1a*-KO mice. Data are expressed as the mean ± SE for 5 animals per group. * *p* < 0.05 vs. 0 Gy and # *p* < 0.05 vs. WT mice.

Asc is synthesized mainly in the liver [14,15] and is a potent antioxidant that reportedly alleviates damages caused by exposure to X-rays [12]. Although it is known that such exposure decreases Asc levels in the adrenal glands of rats [31], this phenomenon has not yet been confirmed in mice. We then measured Asc levels in the plasma, BMCs, liver, and adrenal glands following exposure to X-rays (Figure 2). We found that Asc levels were decreased in the plasma, BMCs, and livers when measured one day following exposure to X-rays, after which the levels began to increase. On the other hand, Asc levels in the adrenal grands were unchanged following exposure to X-rays. The mRNAs for *SVCT1* and *SVCT2*, which act to uptake Asc, were increased in the livers following exposure to X-rays in WT mice (Figure 3). Surprisingly, the *SVCT1* and *SVCT2* mRNAs were undetectable in BMCs both before and after X-ray exposure. In the BMCs of *Akr1a*-KO mice, Asc levels remained low before and after exposure to X-rays. These results suggest that released Asc from the liver is supplied to BMCs to support their protection and regeneration. 

### 3.3. AKr1a-KO Increased Corticosterone Secretion Post-Exposure to X-rays

**Figure 4:**
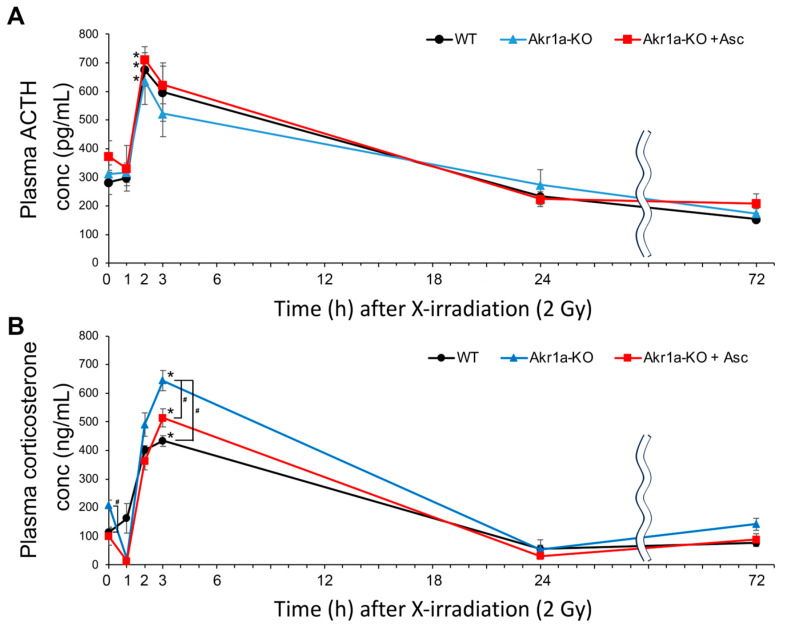
*Akr1a*-KO enhanced the radiation-induced increase in plasma corticosterone levels following exposure to X-rays. ACTH levels (**A**) and corticosterone levels (**B**) in plasma were measured using an ELISA kit in WT and *Akr1a*-KO mice with or without Asc following X-ray exposure at 2 Gy. Data are expressed as the mean ± SE for 5 animals per group. * *p* < 0.05 vs. 0 Gy and # *p* < 0.05 vs. WT mice and *Akr1a*-KO mice supplemented with Asc.

Stress caused by X-rays reportedly promotes ACTH release from pituitary glands, which results in the secretion of corticosterone from adrenal glands [28]. Because corticosterone secretion could impair the recovery of WBCs following exposure to X-rays (Figure 1), we measured the ACTH and corticosterone levels post-exposure in WT and *Akr1a*-KO mice. As reported in a previous study [28], ACTH levels were increased immediately following X-ray exposure in both WT and *Akr1a*-KO mice, but there was no difference in the concentrations between the mouse groups (Figure 4A). Consistent with the increase in ACTH levels, corticosterone levels showed a dramatic increase immediately following exposure to X-rays, followed by a decrease 24 h post-exposure (Figure 4B). In *Akr1a*-KO mice, corticosterone levels were higher compared with those in WT and *Akr1a*-KO mice that were supplemented with Asc before and 3 h following exposure to X-rays, but the levels were decreased to a similar extent one day later. These results suggest that corticosterone released from adrenal glands is associated with Asc levels, and that Asc deficiency adversely affects BMC proliferation.

**Figure 5:**
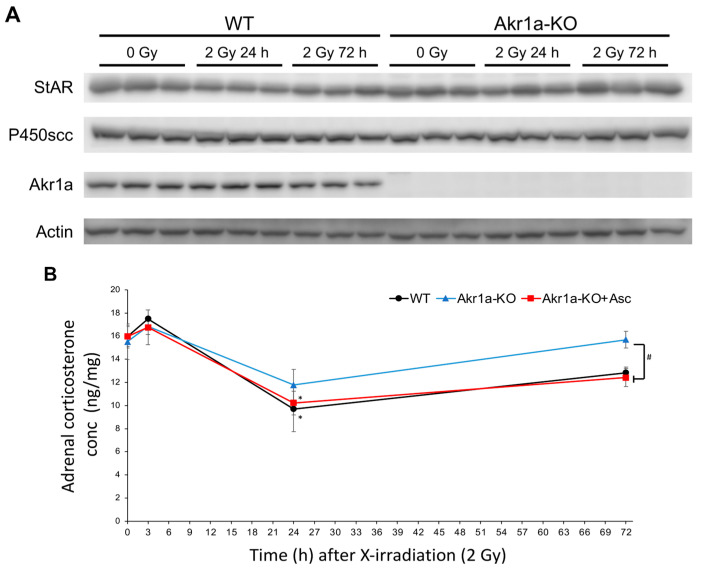
Exposure to X-rays, which did not affect the levels of the primary steroidogenic proteins but transiently decreased corticosterone concentrations. (**A**) The proteins associated with steroid hormone synthesis evaluated via Western blotting in representative blots of StAR, P450_scc_, AKR1A, and Actin. (**B**) Corticosterone levels in adrenal glands measured using an ELISA kit in WT and *Akr1a*-KO mice with or without Asc following X-ray exposure at 2 Gy. Data are expressed as the mean ± SE for 3–5 animals per group. * *p* < 0.05 vs. 0 Gy and # *p* < 0.05 vs. WT mice and *Akr1a*-KO mice supplemented with Asc.

To investigate corticosterone synthesis capacity following exposure to X-rays, we examined the levels of two key proteins in the adrenal glands that are associated with steroid synthesis in WT and *Akr1a*-KO mice before and after exposure. StAR is a steroidogenic acute regulatory protein that is involved in cholesterol transport from the outer to inner mitochondrial membranes [35], and P450_scc_ (CYP11A1) is a cholesterol side-chain cleavage enzyme that regulates the first and rate-limiting steps in the synthesis of steroid hormones [36]. To examine the proteins involved in steroid hormone synthesis following exposure to X-rays, we performed Western blotting on the adrenal glands (Figure 5A). These protein expression levels were unchanged in both WT and *Akr1a*-KO mice before and after X-ray exposure. This result suggests that exposure to X-rays did not affect the protein expression associated with corticosterone synthesis. As shown in Figure 4B, corticosterone levels immediately increased in a few hours and were then decreased one day following exposure to X-rays, which suggests that corticosterone secretion from the adrenal glands had been stimulated by this exposure, but this secretion subsequently declined after 24 h due to excretion. We then evaluated corticosterone levels in the adrenal glands after exposure to X-rays (Figure 5B). Adrenal corticosterone levels had significantly decreased one day following the exposure to X-rays in both WT and *Akr1a*-KO mice. However, the amount of corticosterone in the adrenal glands was lower compared with that in plasma, which indicated that the corticosterone stored in the adrenal glands was low. These results suggest that, following exposure to X-rays, adrenal corticosterone synthesis was initially activated in response to ACTH and then was subsequently reduced, probably through a feedback mechanism, resulting in a decrease in plasma corticosterone levels. 

### 3.4. Asc Treatment Mitigated Radiation-Induced Damage, While Dexamethasone Treatment Enhanced It in Cultured BMCs

**Figure 6:**
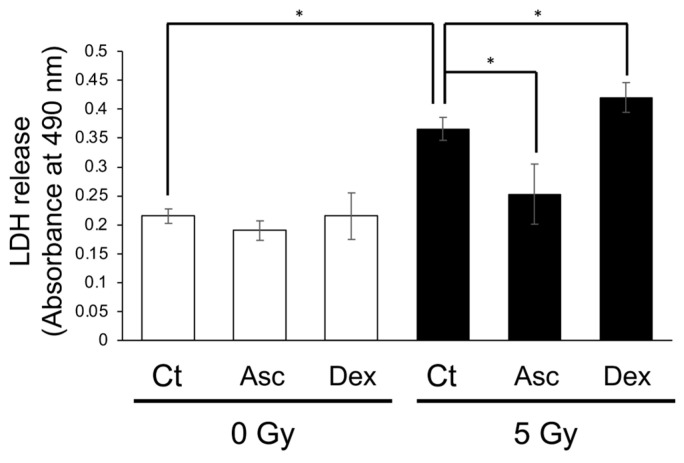
Radiation-induced LDH release from cultured BMCs reduced via Asc treatment but increased via dexamethasone treatment. BMCs were isolated and cultured overnight. The BMCs were treated either with 50 μmol/L Asc or 200 nmol/L dexamethasone (DEX) 2 h before exposure to X-rays at 5 Gy. LDH release from cultured BMCs was measured one day following exposure. Data are expressed as the mean ± SE for 3 samples per group. * *p* < 0.05.

To confirm the effect of Asc and corticosterone on the viability of BMCs following exposure to X-rays, we performed an LDH assay using cultured BMCs from WT mice. Exposure to X-rays at 2 Gy had not damaged the cultured BMCs, and, therefore, we separately administered Asc and dexamethasone 2 h before exposure to 5 Gy. After 24 h of incubation, the medium containing the BMCs was collected and used for the LDH assay (Figure 6). X-ray exposure significantly increased LDH release. Asc treatment reduced the radiation-induced increase in LDH release, while dexamethasone treatment increased it. This result indicated that Asc was protective against radiation-induced damage and that corticosterone had adversely affected the viability of BMCs. 

## 4. Discussion

In the current study, we used *Akr1a*-KO mice, which are defective in synthesizing Asc and show elevations in plasma corticosterone concentrations under ordinary conditions [20]. After exposure to X-rays at 2 Gy, plasma Asc levels were decreased temporarily on the first day, and then tended to recover gradually in all groups of mice (Figure 2). Since supplementation with Asc accelerated the recovery of WBC concentrations, mostly lymphocytes, following the exposure to X-rays in both *Akr1a*-KO and WT mice, low Asc concentrations appeared to be responsible for the delayed recovery in *Akr1a*-KO mice (Figure 1). Following exposure to X-rays, ACTH and corticosterone levels were soon increased in the blood (Figure 4), which appeared to be somewhat lessened in *Akr1a*-KO mice via their Asc status. Asc appeared to be protective against exposure to X-rays also in the primary culture of BMCs (Figure 6), which supports the beneficial action of Asc.

Asc concentrations in both BMCs and blood plasma were transiently decreased on day 1 and then gradually recovered in all groups of mice (Figure 2). The decline in Asc concentrations in BMCs has also been reported in rats following exposure to X-rays, but no change has been observed in the blood levels of Asc [31]. In our study, WBCs were decreased similarly in all experimental groups, but the recovery was delayed in *Akr1a*-KO mice at one month following their exposure to X-rays (Figure 1). Cells either with a low degree of differentiation or those in proliferation seem to be more susceptible to radiation damage. Therefore, a delay in the recovery of WBC concentrations appears to reflect a degree of selective damage to the less-differentiated progenitor cells such as hematopoietic stem cells (HSCs) in BMCs. Since supplementation with Asc rescued the abnormality of the *Akr1a*-KO mice to an extent similar to that in the WT mice (Figure 1), we assumed that Asc either protected the BMCs from oxidative damage or stimulated the hematopoietic process. In fact, a recent clinical study has reported that intravenous administration of Asc (50 mg/kg/day, divided into three doses given on days 1–14) to patients undergoing allogeneic HSC transplant is safe and reduces non-relapse mortality, resulting in the improvement of overall survival [37]. The intravenous administration of mesenchymal stem cells overexpressing extracellular superoxide dismutase (*SOD3*) to the mice after 9-Gy γ-ray irradiation reportedly improved their survival [38]. The results imply that SOD3 was produced by the homed cells and protected the irradiated tissues including bone marrow through the scavenging of superoxide by SOD3. Since Asc also effectively scavenges radicals including superoxide [7,8], it is thought that HSCs were protected from the cytotoxic effects of oxygen radicals by Asc in our study.

The adrenal gland is the first organ that was discovered to be enriched in Asc [22], but the roles of Asc have long been debated. Asc reportedly stimulates steroidogenesis in the adrenal glomerular zone of rats [23]. Genes responsible for steroidogenesis, including those encoding P450_scc_, 3-hydroxysteroid dehydrogenase type 1, and aromatase, are induced by Asc in human choriocarcinoma cells [24]. Consistent with these reports, the knockout of *SVCT2* impairs adrenal chromaffin cells and decreases plasma levels of corticosterone as well as tissue levels of adrenalin/noradrenalin [39]. In the present study, however, the levels of corticosterone in the plasma of *Akr1a*-KO mice were originally high and were transiently increased following exposure to X-rays, despite levels of Asc that were quite low in the plasma and adrenal glands regardless of exposure (Figure 2). Similar phenomena have been found in other animal models with Asc deficiency as follows. Rats with an osteogenic disorder (ODS), which presents a hereditary defect in Asc-synthesizing ability due to a mutation of the *Gulo* gene, show higher plasma and adrenal levels of corticosterone [40]. Guinea pigs, which also carry a defect in the *Gulo* gene, show higher levels of glucocorticoid in the blood due to Asc deficiency [41]. Thus, high Asc levels seem to rather suppress glucocorticoid levels in the blood of these animal species, and it is unlikely that Asc supports steroidogenesis in the adrenal glands.

Whole-body exposure to X-rays causes stress that stimulates the pituitary–adrenal axis. As a result, exposure to X-rays promotes the secretion of ACTH, which leads to a release of adrenocortical hormones, mainly corticosterone, in mice [28]. Given the rapid secretion of ACTH from pituitary glands [29] and the short half-life of corticosterone in the blood of only a few hours in humans [42], lower corticosterone concentrations after one day would reflect fatigue in the steroidogenic ability of the cells in adrenal glands. Protection of the lower portion of the body by a lead shield causes a greater release of corticosterone and adrenal Asc in comparison with that under total-body irradiation in rats [2]. These observations demonstrate that exposure to X-rays first stimulates the release of ACTH from pituitary glands, which leads to the stimulation of corticosterone production [29]. ACTH also stimulates the release of Asc from the liver and adrenal glands in rats [43]. Therefore, Asc recovery in plasma following X-ray exposure could be attributable to recruitment from the liver, which is the main organ that produces Asc in rodents [14]. A following study has shown that ACTH also stimulates the release of Asc from the adrenal glands in humans [30]. Based on these findings, we know that upon exposure to X-rays, ACTH release from pituitary glands then stimulates not only corticosterone secretion but also Asc release from both the adrenal glands and the liver.

Anti-inflammation is the well-established function of corticosterone, and the inhibition of a transcriptional regulatory factor NF-κB, which dominantly induces expression of pro-inflammatory genes, is one of its mechanisms [44]. On the other hand, BMCs are prone to apoptosis under exposure to corticosterone as well as under exposure to X-rays [5,45]. Glucocorticoid stimulates energy metabolism to cope with stress conditions, but, at the same time, it adversely affects BMCs, notably in lymphocytes [46,47]. Glucocorticoid is known to increase ROS in part by suppressing the function of Nrf2, which is a key transcriptional activator for the expression of some of the antioxidant genes [48,49]. Since the replenishment of Asc leads to a decreased glucocorticoid response in humans and in animal models after exposure to a psychological or physical stressors [50], Asc likely acts to suppress the injurious effects of the glucocorticoid that is released under stress conditions. Because Asc is a potent antioxidant, scavenging oxygen radicals with electron donation [7,8], the primary action of Asc appears to be that of protecting BMCs against ROS that are produced by ionizing radiation and by corticosterone response. Since Asc exerts pleiotropic functions, it is also possible that Asc stimulates the hematopoietic process by supporting enzymatic reactions, such as prolyl hydroxylase, or by epigenetically regulating gene expression [51]. Considering the cytotoxic action of corticosterone, the early recovery of WBCs in ADX mice following exposure to X-rays might be associated with an absence of cytotoxic effects of plasma corticosterone.

## 5. Conclusions

**Figure 7:**
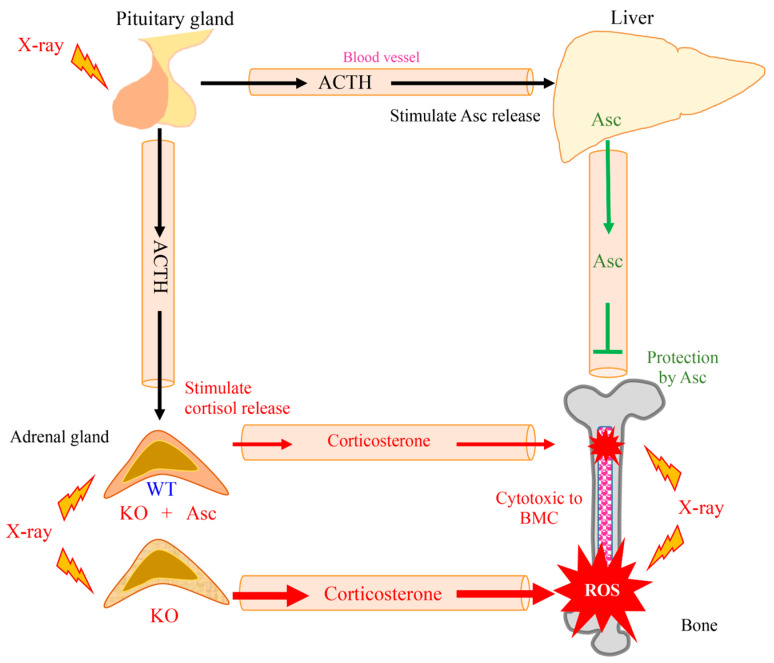
Schematic diagram outlining the results of this study.

In this study, Asc deficiency delayed the recovery of WBCs in *Akr1a*-KO mice following exposure to a permissive dose of X-rays, which are known to increase ROS production via the interaction of oxygen molecules, but also trigger the secretion of corticosterone through the stimulation of ACTH released from pituitary glands. Asc deficiency in the *Akr1a*-KO mice elevated corticosterone levels, and oxidative damage was increased via such suppression of antioxidant enzyme expression, which appears to also be partially responsible for aggravating the damage to BMCs. It is conceivable that physiological levels of Asc alleviate the damage to BMCs through their antioxidant capacity against ROS that are produced by exposure to X-rays and via the attendant cytotoxic action of corticosterone (Figure 7). 

## Data Availability

The data underlying this article are available in the article and Supplementary Materials.

## Supplementary Materials

The following supporting information can be downloaded at https://www.mdpi.com/article/10.3390/antiox13020152/s1. Figure S1: Changes in count of granulocytes following X-ray irradiation; Figure S2: Liver and kidney damages not observed after Xray irradiation in WT and *Akr1a*-KO mice; Table S1: Primer sequence for quantitative-RT PCR.

## Abbreviations

ACTH	adrenocorticotropic hormone
ADX	adrenalectomy
AKR	aldo-keto reductase
AKR1A	aldehyde reductase
*Akr1a*	gene encoding adehyde reductase
AKR1B	aldose reductase
ALT	alanine aminotransferase
Asc	ascorbic acid
AST	aspartate aminotransferase
BMCs	bone marrow cells
BUN	blood urea nitrogen
CRE	creatinine
*Gulo*	gene encoding L-gulono-γ-lactone oxidase
KO	knockout
LDH	lactate dehydrogenase
ROS	reactive oxygen species
SE	standard error
WBCs	white blood cells
WT	wild-type
