# Supplementary material for: Ascorbic Acid Protects Bone Marrow from Oxidative Stress and Transient Elevation of Corticosterone Caused by X-ray Exposure in Akr1a-Knockout Mice
Source: Antioxidants (Basel). 2024 Jan 25;13(2):152. doi: 10.3390/antiox13020152 (PMC10886414; doi:10.3390/antiox13020152)
Supplement: Supplementary file 1 [file antioxidants-13-00152-s001.zip › Supplementary Figure S1.pptx]

## Slide 1
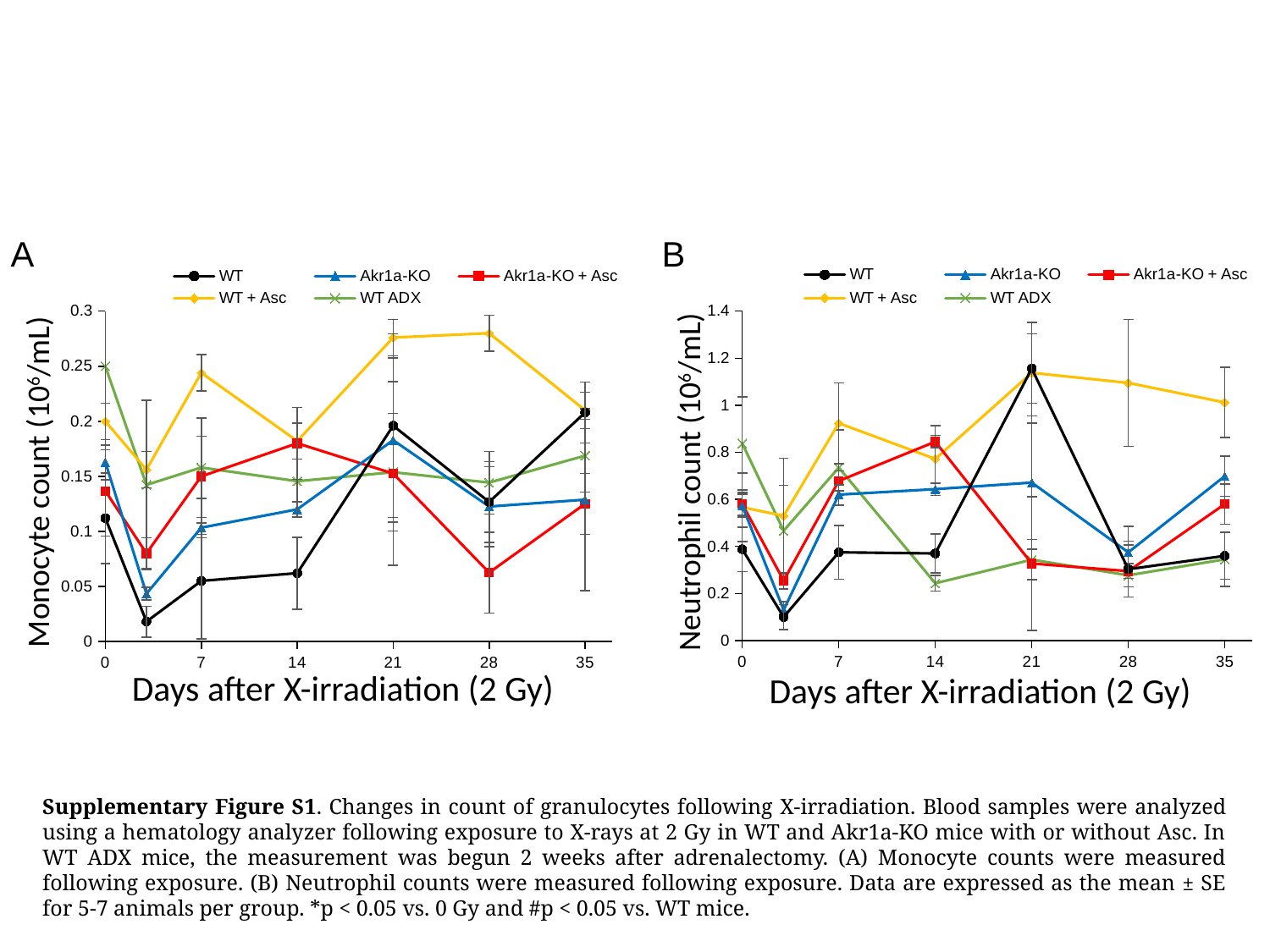

A
 B
### Chart
| Category | WT | Akr1a-KO | Akr1a-KO + Asc | | |
|---|---|---|---|---|---|
### Chart
| Category | WT | Akr1a-KO | Akr1a-KO + Asc | | |
|---|---|---|---|---|---|Monocyte count (106/mL)
Neutrophil count (106/mL)
Days after X-irradiation (2 Gy)
Days after X-irradiation (2 Gy)
Supplementary Figure S1. Changes in count of granulocytes following X-irradiation. Blood samples were analyzed using a hematology analyzer following exposure to X-rays at 2 Gy in WT and Akr1a-KO mice with or without Asc. In WT ADX mice, the measurement was begun 2 weeks after adrenalectomy. (A) Monocyte counts were measured following exposure. (B) Neutrophil counts were measured following exposure. Data are expressed as the mean ± SE for 5-7 animals per group. *p < 0.05 vs. 0 Gy and #p < 0.05 vs. WT mice.
