# Supplementary material for: Ascorbic Acid Protects Bone Marrow from Oxidative Stress and Transient Elevation of Corticosterone Caused by X-ray Exposure in Akr1a-Knockout Mice
Source: Antioxidants (Basel). 2024 Jan 25;13(2):152. doi: 10.3390/antiox13020152 (PMC10886414; doi:10.3390/antiox13020152)
Supplement: Supplementary file 1 [file antioxidants-13-00152-s001.zip › Supplementary Figure S2.pptx]

## Slide 1
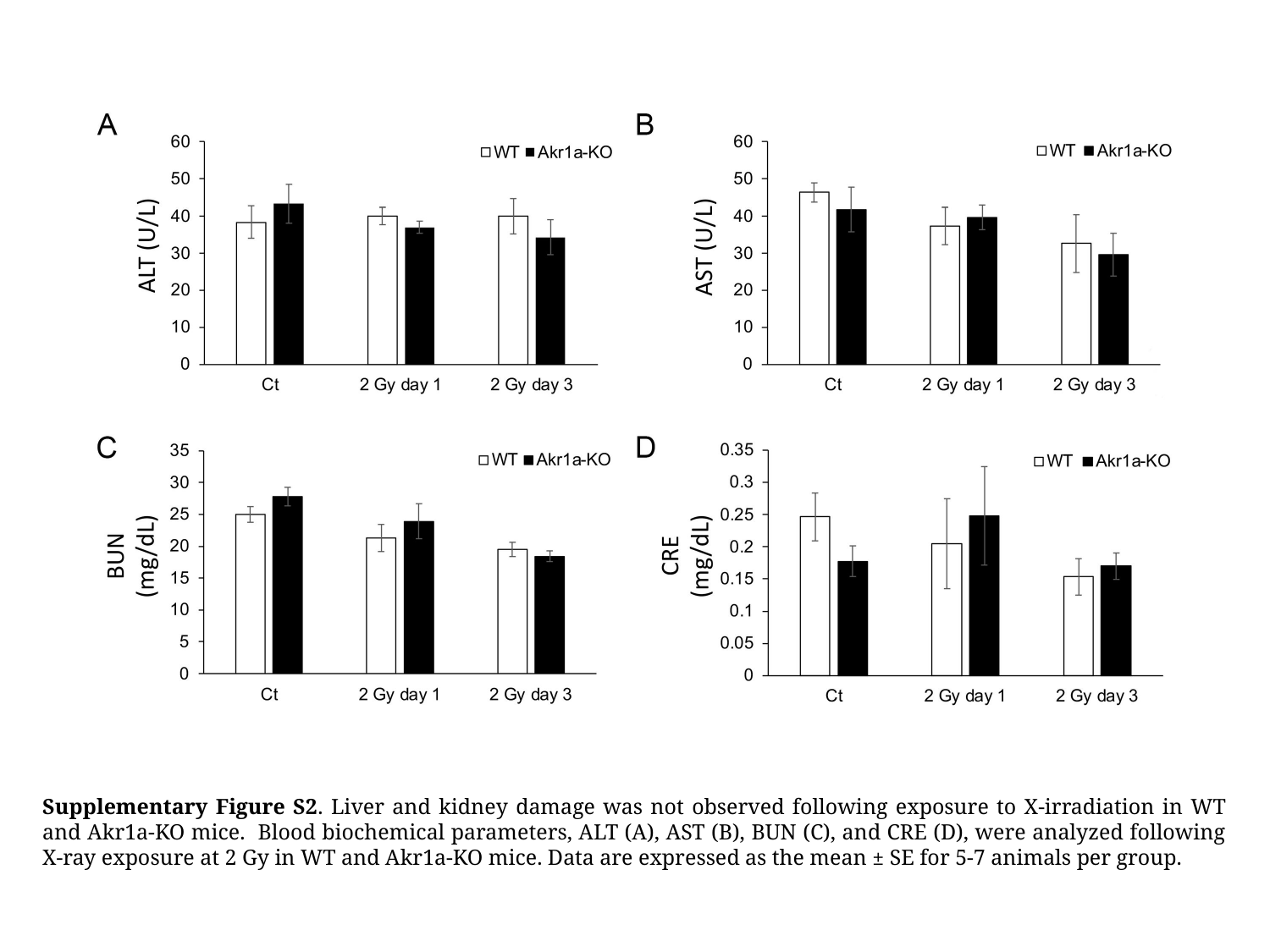

Supplementary Figure S2. Liver and kidney damage was not observed following exposure to X-irradiation in WT and Akr1a-KO mice. Blood biochemical parameters, ALT (A), AST (B), BUN (C), and CRE (D), were analyzed following X-ray exposure at 2 Gy in WT and Akr1a-KO mice. Data are expressed as the mean ± SE for 5-7 animals per group.
