# Supplementary material for: Ascorbic Acid Protects Bone Marrow from Oxidative Stress and Transient Elevation of Corticosterone Caused by X-ray Exposure in Akr1a-Knockout Mice
Source: Antioxidants (Basel). 2024 Jan 25;13(2):152. doi: 10.3390/antiox13020152 (PMC10886414; doi:10.3390/antiox13020152)
Supplement: Supplementary file 1 [file antioxidants-13-00152-s001.zip › Supplementary Table S1.pptx]

## Slide 1
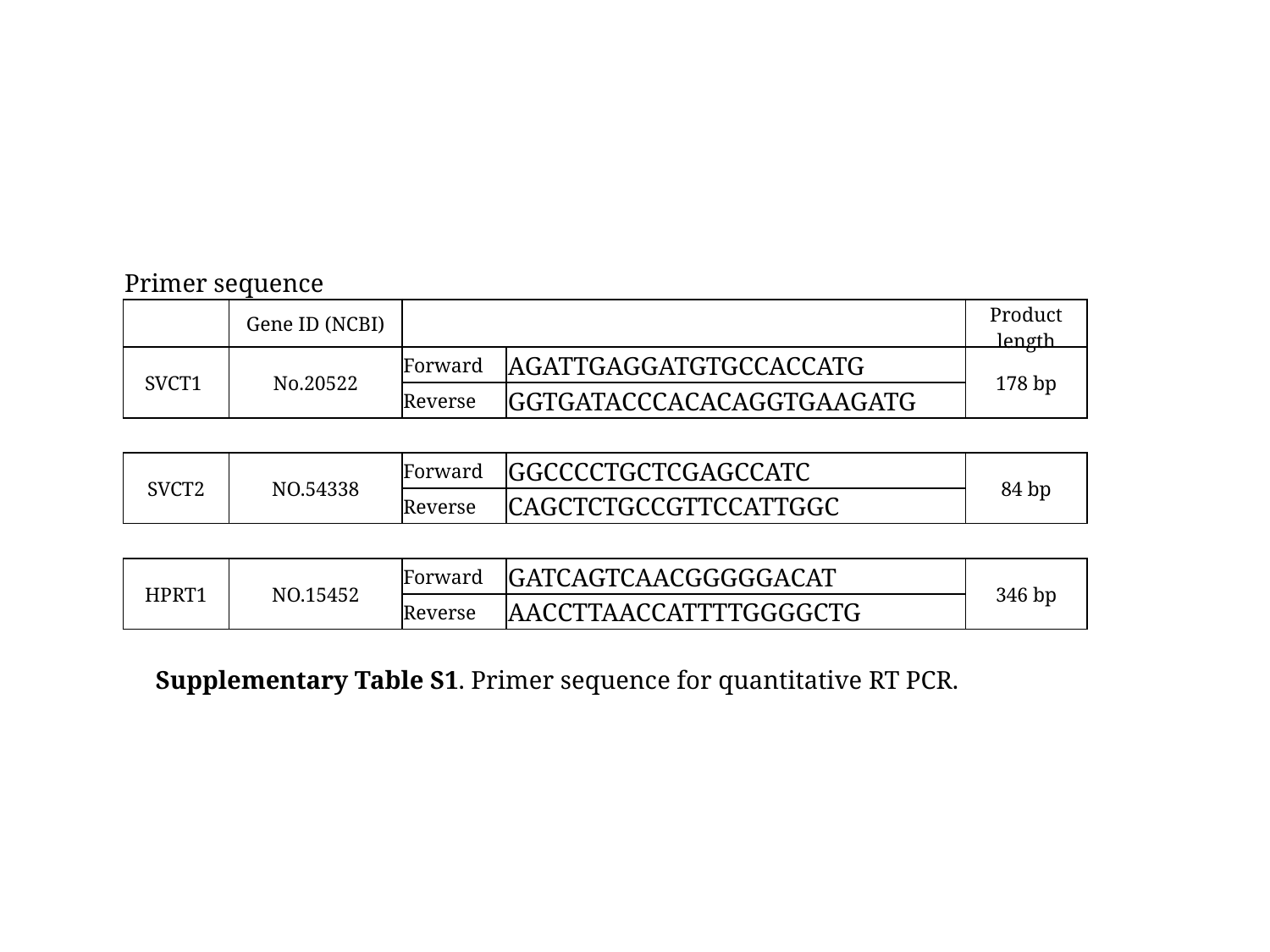

| Primer sequence | | | | | | | |
| --- | --- | --- | --- | --- | --- | --- | --- |
| | Gene ID (NCBI) | | | | | | Product length |
| SVCT1 | No.20522 | Forward | AGATTGAGGATGTGCCACCATG | | | | 178 bp |
| | | Reverse | GGTGATACCCACACAGGTGAAGATG | | | | |
| | | | | | | | |
| SVCT2 | NO.54338 | Forward | GGCCCCTGCTCGAGCCATC | | | | 84 bp |
| | | Reverse | CAGCTCTGCCGTTCCATTGGC | | | | |
| | | | | | | | |
| HPRT1 | NO.15452 | Forward | GATCAGTCAACGGGGGACAT | | | | 346 bp |
| | | Reverse | AACCTTAACCATTTTGGGGCTG | | | | |
Supplementary Table S1. Primer sequence for quantitative RT PCR.
